# The relationship between hypoxia and Alzheimer’s disease: an updated review

**DOI:** 10.3389/fnagi.2024.1402774

**Published:** 2024-07-17

**Authors:** Borui Tao, Wei Gong, Chengyuan Xu, Zhihui Ma, Jinyu Mei, Ming Chen

**Affiliations:** ^1^Department of Pharmacology, School of Basic Medical Sciences, Anhui Medical University, Hefei, China; ^2^The First Clinical Medical College, Anhui Medical University, Hefei, China; ^3^Department of Otorhinolaryngology Head and Neck Surgery, The Second Affiliated Hospital of Anhui Medical University, Hefei, China

**Keywords:** Alzheimer’s disease, hypoxia, HIF-1α, oxidative stress, Aβ, mitochondrial dysfunction, inflammation, tau

## Abstract

Alzheimer’s disease (AD) is one of the most common neurodegenerative diseases, and the most prevalent form of dementia. The main hallmarks for the diagnosis of AD are extracellular amyloid-beta (Aβ) plaque deposition and intracellular accumulation of highly hyperphosphorylated Tau protein as neurofibrillary tangles. The brain consumes more oxygen than any other organs, so it is more easily to be affected by hypoxia. Hypoxia has long been recognized as one of the possible causes of AD and other neurodegenerative diseases, but the exact mechanism has not been clarified. In this review, we will elucidate the connection between hypoxia-inducible factors-1α and AD, including its contribution to AD and its possible protective effects. Additionally, we will discuss the relationship between oxidative stress and AD as evidence show that oxidative stress acts on AD-related pathogenic factors such as mitochondrial dysfunction, Aβ deposition, inflammation, etc. Currently, there is no cure for AD. Given the close association between hypoxia, oxidative stress, and AD, along with current research on the protective effects of antioxidants against AD, we speculate that antioxidants could be a potential therapeutic approach for AD and worth further study.

## 1 Introduction

Alzheimer’s disease (AD) is one of the most common neurodegenerative diseases, and the most prevalent form of dementia. It usually manifests as a gradual decline in episodic memory and cognitive abilities, leading to impairments in language, visuospatial skills, and often behavioral disturbances like apathy, aggression, and depression (Høgh, 2017; Porsteinsson et al., 2021). The main neuropathological criteria for the diagnosis of AD are extracellular amyloid-beta (Aβ) plaque deposition and intracellular accumulation of highly hyperphosphorylated Tau protein as neurofibrillary tangles (Long and Holtzman, 2019). As the population ages, the incidence of AD continues to rise (Weller and Budson, 2018). According to Alzheimer’s Disease International the prevalence of dementia is about 50 million people worldwide, and is predicted to more than triple by 2050 as the population ages (Lane et al., 2018; Scheltens et al., 2021). Over the next few years, there’s an anticipated spike in dementia prevalence, particularly in low and middle income countries, aligning with the rising incidence of cardiovascular disease, hypertension, and diabetes, while on the contrary, emerging evidence indicates a decline in dementia incidence in high-income countries, although the evidence supporting a decrease in prevalence is less convincing (Lane et al., 2018; Scheltens et al., 2021). In the United States, unpaid dementia caregiving was valued at US$346.6 billion in 2023 and may exceed US$600 billion in 2050 which results in great societal burden (Lane et al., 2018; Alzheimer’s Association, 2024). AD is a heterogeneous disease with a complicated pathophysiology. Mounting evidence show that AD is 60%∼80% dependent on heritable factors and there are several hypothesizes such as the amyloid and neuro-inflammation hypothesis (Liu et al., 2019). Hypoxia is also believed to have a tight connection with AD. Our brain consumes 20% of the oxygen and maintains a continually active state relying on the oxygen (Bailey, 2019). As a result of the high energy-consumption of the brain, it is more likely to be influenced by hypoxia than any other organ. Neurons, as the basic functional unit of the brain, are also likely to be influenced by hypoxia as they contain low levels of glutathione which plays crucial roles in the antioxidant defense system and the maintenance of redox homeostasis in neurons (Aoyama, 2021). Since the 19th century, people realized that hypoxia can lead to neurological consequences (Burtscher et al., 2021). There are evidences showing that hypoxia has a tight connection with AD. Studies show that the risk of AD increases a lot after persistent systemic hypoxia or stroke (Vijayan and Reddy, 2016; Sriram et al., 2022) and reduced oxygen supply has also been observed in both AD pathology and the aging process (Adeyemi et al., 2021). As the underlying molecular mechanisms connecting hypoxia with AD is still unclear, the involvement of kynurenine pathway has gained interest. Tryptophan (Trp) is an essential amino acid as it cannot be produced in human body and it is a precursor to a number of metabolites like serotonin, melatonin, and niacin as well as neurotransmitters (Mohapatra et al., 2021). The kynurenine pathway is one of the three major pathways of Trp metabolism which metabolizes 90% of Trp into kynurenic acid, xanthurenic acid, picolinic acid, quinolinic acid, and nicotinamide adenine dinucleotide (Cervenka et al., 2017; Doifode et al., 2021). Studies have found that the several metabolites of kynurenine pathway including quinolinic acid, kynurenine and 3-hydroxykynurenine are associated with AD, due to their involvement in excitotoxic neurotransmission, oxidative stress, uptake of neurotransmitter, amyloid aggregation, and inflammation (Wang et al., 2015; Venkatesan et al., 2020; Sharma et al., 2022). Studies also found that hypoxia can induce the increase of Trp production thus leading to more metabolites of kynurenine pathway, thus suggesting a connection between hypoxia and AD (Mohapatra et al., 2021). However, there are researches showing that the increase of Trp in hypoxia is due to the decrease of kynurenine pathway function suggesting that Trp catabolites are not key of factors in the pathophysiology of AD (Mohapatra et al., 2021; Almulla et al., 2022). There is also evidence showing that hypoxia is related with AD. Chronic intermittent hypoxia (CIH) is a feature of obstructive sleep apnea (OSA). Recent studies on OSA compared the serum levels of Aβ proteins and tau proteins in 46 cognitively normal OSA patients and 30 healthy controls: the results showed that patients with OSA had significantly higher median serum levels of Aβ_40_, Aβ_42_ and total tau than controls. One study also found that Aβ level are associated with the changes in sleep architecture, specifically, rapid eye movement sleep was negatively correlated with Aβ proteins. Another study on APP/PS1 mice (an animal model of AD) examined the effects of CIH on cognition and hippocampal function and found that CIH induced long-term potentiation dysfunction of the hippocampus in APP/PS1 mice as they found the decrease of N-methyl-D-aspartic acid receptor (NMDAR) NR1 subunit and postsynaptic density 95 (PSD95) in the hippocampus of APP/PS1 mice after CIH treatment (Li and Ye, 2024). NMDAR is a type of ionotropic glutamate receptor found in nerve cells while PSD95 is a scaffolding protein found in the post-synaptic density of neurons. They are both crucial for synaptic plasticity, learning, and memory processes in the brain. These results suggest that CIH is related to the AD (Bhuniya et al., 2022).

As the accurate pathogenesis of AD is still unknown, a good understanding of the relationship between hypoxia and AD can help us know more about this disease and help discover potential therapeutic approaches for it. In this review, we focus on the relationship between hypoxia-inducible factors-1α (HIF-1α) with AD as well as the link between oxidative stress and AD.

## 2 HIF-1α is an essential factor in AD onset

### 2.1 Structure and function of HIF-1α

**FIGURE 1:**
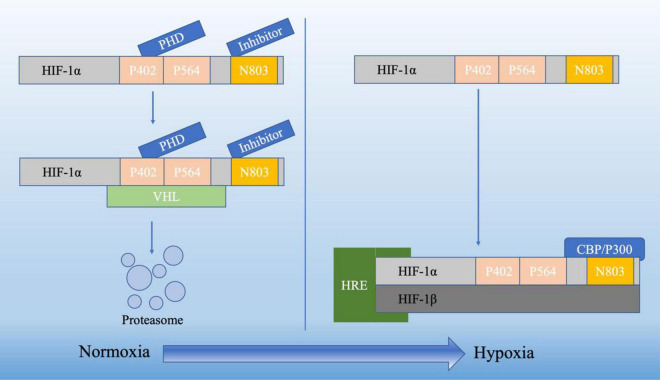
The HIF-1α undergoes distinct cleavage processes in normoxia and hypoxia. In normoxia, hydroxylation of two proline residues and acetylation of a lysine residue within the oxygen-dependent degradation domain prompt its binding to the VHL E3 ligase complex, initiating degradation through the ubiquitin-proteasome pathway. Conversely, in hypoxia, the HIF-1α subunit attains stability and engages with coactivators like CBP/P300, orchestrating the regulation of gene expression.

Hypoxia-inducible factors (HIFs) are transcription factors consisting of α and β subunits that regulate cellular reactions to low oxygen levels. The latter is a constructive subunit which forms a heterodimeric complex with the former, while the former is an oxygen-sensitive subunit, thus, the transcriptional activity of HIF-1 is primarily regulated by the levels of HIF-1α protein (Forsythe et al., 1996; Zagórska and Dulak, 2004; Figure 1).

Three isoforms of HIF-α (HIF-1α, HI F-2α, HI F-3α) have been identified. Among the three types of HIF-α isoforms, HIF-1α is involved in the acute hypoxic response associated with erythropoietin, whereas HIF-2α is associated with the response to chronic hypoxia (Xie et al., 2019). Under normal oxygen conditions, HIF-1α undergoes degradation through a process involving the von Hippel-Lindau (VHL) protein. Prolyl hydroxylase enzymes (PHDs) are a group of enzymes which can be found in various tissues and cells throughout the body, including the liver, kidneys, and heart. They are involved in the modification of proteins, specifically in the hydroxylation of proline residues. In normoxia, PHDs are active and hydroxylate specific proline residues on HIF-1α at P402 and P564. This hydroxylation marks HIF-1α for recognition by the VHL protein, which is part of an E3 ubiquitin ligase complex. Upon binding to hydroxylated HIF-1α, the VHL complex ubiquitinates HIF-1α. This ubiquitination signals for the proteasomal degradation of HIF-1α, preventing its accumulation and subsequent activation of HIF-1. This process is a key regulatory mechanism that ensures HIF-1α is degraded under normoxic conditions, maintaining cellular homeostasis in the presence of sufficient oxygen (Yu et al., 2001; Semenza, 2007; Figure 1). Conversely, under hypoxic conditions, HIF-1 levels can quickly increase in order to adapt from anoxic condition (Wang et al., 1995). In low oxygen condition, PHDs are less active: as a result, HIF-1α is not hydroxylated as extensively. With reduced hydroxylation and degradation, HIF-1α accumulates in the cytoplasm, where it stabilizes and can translocate into the cell nucleus. In the nucleus, HIF-1α forms a complex with HIF-1β. This heterodimeric complex is the active form of HIF-1. The HIF-1 complex binds to specific DNA sequences called Hypoxia-Response Elements (HREs) in the promoter regions of target genes (Yang C. et al., 2021). CREB-binding protein (CBP) and p300 are related transcriptional coactivators that play important roles in regulating gene expression by interacting with a variety of transcription factors. They possess histone acetyltransferase activity, which allows them to modify chromatin structure and promote transcription (Wu et al., 2013). When HIF-1α is stabilized under hypoxic conditions, the C-terminal transactivation domain forms a complex with CBP/p300. The latter then acetylates specific lysine residues on HIF-1α, enhancing its transcriptional activity. This acetylation event facilitates the recruitment of additional transcriptional machinery, leading to the transcription of genes involved in cellular adaptation to low oxygen levels (Figure 1; Dames et al., 2002; Wu et al., 2013). Finally, under hypoxic conditions, HIF-1α together with other molecular mediators like peroxisome proliferator-activated receptor γ coactivator α (PGC-1α), c-MYC (a protein plays a crucial role in regulating cell growth, proliferation and apoptosis), SIRT1 (a protein involved in regulating various cellular processes such as aging, DNA repair, metabolism, and stress response), and AMPK (an enzyme that plays a crucial role in cellular energy homeostasis which is activated in response to low cellular energy levels) become activated and function as transcription factors, regulating the expression of genes involved in various adaptive responses (Ham and Raju, 2017). HIF-1 promotes the cellular adaptation to hypoxia by activating genes that enhance oxygen delivery. For example, VEGF, acting as the downstream target gene of HIF-1α, is crucial in controlling angiogenesis (formation of new blood vessels). Research indicates that the HIF-1α/VEGF pathway participates in various pathophysiological processes, including inflammation, ischemia-reperfusion injury, oxidative stress, and other conditions associated with angiogenesis or vascular remodeling as well as tumor immunity (Palazon et al., 2017; Liu et al., 2018; Lin et al., 2019; Chen et al., 2022). HIF-1α is also involved in the acute hypoxic response associated with erythropoiesis (Sala et al., 2018; Xie et al., 2019; Hirota, 2021). Additionally, it stimulates glycolysis, a process that does not rely on oxygen, providing an alternative energy source when oxygen availability is limited (Cheng et al., 2014; Wang et al., 2021).

### 2.2 HIF-1α in Alzheimer’s disease: friend or foe?

Amyloid-beta is formed by the sequential cleavage of amyloid precursor protein (APP) by β-secretases (BACE1) as well as γ-secretases (Sun et al., 2017) and its accumulation in brain tissue is now acknowledged as the major pathogenic event in AD (Zou et al., 2020). BACE1 serves as a pivotal enzyme in APP processing, linked to the generation of the membrane-bound C-terminal fragment C99 (APP-C99) and its production. Numerous studies have highlighted the involvement of BACE1 regulation in AD pathogenesis, including Aβ accumulation and memory impairment associated with Aβ (Ohno et al., 2004; Laird et al., 2005). γ-Secretase is a macromolecular complex that contains four essential subunits: anterior pharynx-defective 1 (Aph1), nicastrin (NCT), presenilin enhancer 2 (Pen-2), and its catalytic core presenilin (PS) (Yang G. et al., 2021). Aβ exists in a variety of species, including monomers, soluble oligomers, protofibrils, and insoluble fibrils, which are eventually deposited as senile plaques (Huynh et al., 2017; Chai et al., 2021). Specifically, Aβ peptides can transform structurally from monomers into β-stranded fibrils via multiple oligomeric states. Structured oligomers among different Aβ species are suggested to be more toxic than fibrils and the identification of Aβ oligomers has proven challenging due to their diversity and instability (Lee et al., 2017). Research on the relationship between hypoxia and Aβ generation has been conducted for a long time, however, the specific mechanisms remain unclear. We suggest that HIF-1α may connect them. A number of studies highlight that hypoxia, through the mediation of HIF-1α, leads to an increase in BACE1 expression and contributes to elevated Aβ production which is considered the driving force of AD according to the amyloid hypothesis, the most accepted theory for AD pathogenesis (Zhang et al., 2007; Guglielmotto et al., 2009). *In vitro* and *in vivo* studies indicate that hypoxia up-regulates BACE1 expression through a biphasic mechanism both *in vitro* and *in vivo*. The early post-hypoxic upregulation of BACE1 depends on the production of reactive oxygen species (ROS) caused by the sudden interruption of the mitochondrial electron transport chain, which will be discussed in the next part, while the late expression of BACE1 is attributed to the activation of HIF-1α (Guglielmotto et al., 2009). A study by Zhang et al. (2007) further elucidated that overexpression of HIF-1α leads to elevated levels of both BACE1 mRNA and protein, while when HIF-1α is downregulated, BACE1 levels decrease. Meanwhile, this study also shows that hypoxia treatment after HIF-1α activation does not further increase the expression of BACE1, suggesting that hypoxia-induced BACE1 expression is predominantly mediated by HIF-1α (Zhang et al., 2007). In addition, HIF-1α is able to bind and activate γ-secretase, thus promoting the production of Aβ under hypoxic conditions and reduced blood flow in the brain (Alexander et al., 2022). Another study also showed that HIF-1α activated BACE1 and γ-secretase through different ways: HIF-1α transcriptionally upregulates BACE1 and non-transcriptionally activates γ-secretase for Aβ production (Alexander et al., 2022). Moreover, HIF-1α also plays a crucial role in regulating Aβ generation under the influence of other environmental factors like high-glucose. Specifically, studies have indicated that BACE1 localizes within the lipid raft, and alterations in cholesterol levels within these rafts could impact BACE1 function, consequently affecting Aβ generation. This implies that modifications to lipid rafts induced by hyperglycemia might serve as a potential initiator of AD pathogenesis. Under high glucose conditions, increased levels of ROS trigger the activation of HIF-1α and liver X receptor α (LXRα) which is a key factor regulating intracellular cholesterol. This stimulation leads to the reorganization of lipid rafts, thereby enhancing the production of Aβ mediated by BACE1 (Lee et al., 2016).

**FIGURE 2:**
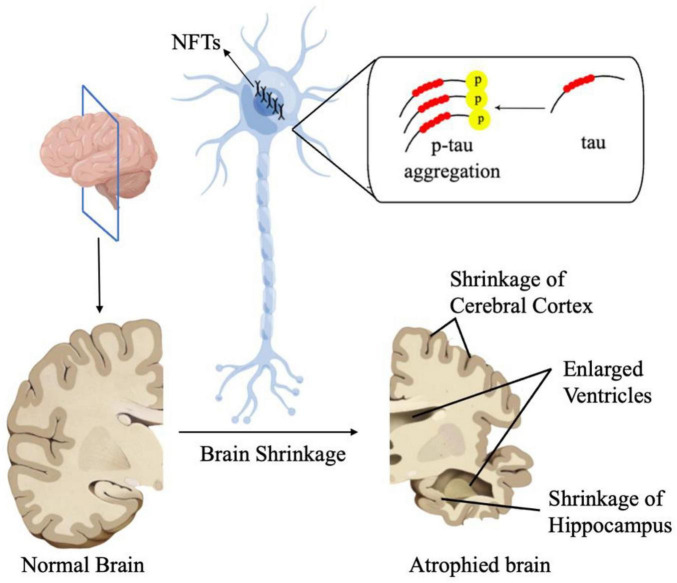
In AD, Tau proteins undergo pathological aggregation, leading to the formation of NFTs. These NFT plaques are one of the characteristic features of AD.

Tau protein, encoded by MAPT on chromosome 17Q21, is a microtubule-associated protein (Lou et al., 2023). Hyperphosphorylation of Tau leads to its pathological aggregation, thus eventually promoting the formation of intracellular neurofibrillary tangles (NFTs). These NFTs, along with Aβ plaques, are characteristic features of AD (Scheltens et al., 2021; Figure 2). There is evidence showing that under intermittent hypoxia conditions, the levels of Tau protein in the serum increase, indicating a close relationship between hypoxia and Tau protein (Bhuniya et al., 2022). However, the specific mechanism of how hypoxia affects tau metabolism remains unclear. A recent study suggested HIF-1α may play a vital role in tau pathology. Under conditions of chronic hypoxia, HIF-1α leads to a deficiency in leucine carboxyl methyltransferase 1 (LCMT1) and protein phosphatase 2A (PP2A), thereby mediating the abnormal hyperphosphorylation of Tau protein (Lei et al., 2022). LCMT1 is an enzyme that plays a role in the methylation of the carboxyl group on leucine residues in proteins. The specific function of LCMT1 includes regulating the activity of PP2A, a critical enzyme involved in the regulation of various cellular processes such as cell division, signal transduction, and metabolism. LCMT1-mediated methylation of PP2A catalytic subunits enhances the activity of PP2A, thereby affecting its ability to dephosphorylate target proteins and modulating cellular signaling pathways (Lei et al., 2022). The methylation activity of LCMT1 can influence the phosphorylation levels of Tau protein, thereby regulating the biological functions of Tau. Specifically, the methylation activity of LCMT1 may contribute to maintaining the normal physiological state of Tau protein, preventing its excessive phosphorylation. However, if LCMT1 function is impaired or disrupted, it may lead to the abnormal phosphorylation of Tau protein, thus contributing to the pathogenesis of neurological disorders, such as AD (Sontag et al., 2013, 2014). Another study conducted on Sprague-Dawley rats shows that there is a significant increase in the phosphorylated PP2A and a significant decrease in the methylated PP2A levels in the rats’ hippocampus after hypoxia treatment. Combined with the elevated tau protein levels in rats, it can be concluded that hypoxia can lead to inactivation of PP2A, resulting in hyperphosphorylation of tau protein and memory deficits (Zhang et al., 2014). However, there are also studies showing that HIF-1α plays a protective role in tau pathology in AD. Glucose transporters (GLUTs) are proteins that facilitate the transport of glucose across cell membranes. They play a crucial role in glucose uptake, particularly in cells that rely heavily on glucose for energy, such as neurons. According to Liu et al. (2008), decreased brain levels of HIF-1α in AD patients were linked to the downregulation of GLUT-1 and GLUT-3 compared to age-matched controls. This impedes glucose uptake and metabolism, ultimately resulting in reduced O-GlcNAcylation and subsequent hyperphosphorylation of tau (Liu et al., 2008). T-2 toxin is a type A trichothecene mycotoxin produced by certain *Fusarium* species. It has been regarded as a neurotoxin as it can enter the brain through the blood-brain barrier. After entering the brain, T-2 toxin can cause further damage by triggering oxidative stress, neuroinflammation, and even apoptosis and it can also induce the rise of phosphorylated tau protein. A study has found that T-2 toxin can induce the expression of hyperphosphorylated tau (Zhao et al., 2024). In Zhao et al.’s (2024) study, they found the level of hyperphosphorylated tau induced by T-2 toxin increased when HIF-α signaling was inhibited. This suggested that HIF-1α played a protective role in the T-2 toxin-induced expression of hyperphosphorylated tau (Zhao et al., 2024). Taken together, we have sufficient reasons to believe that HIF-1α is associated with tau pathology in AD, however, the specific mechanism remains unclear and requires further research.

Inflammation is a pathological process characterized by injury or destruction of tissues. There is a substantial amount of evidence indicating that both neuro-inflammation and the inflammation in periphery are also a significant factor in the development of AD (Heneka et al., 2015; Ozben and Ozben, 2019; Xie et al., 2021; Lou et al., 2023). Zhao et al. (2021) found that HIF-1α is implicated in the inflammatory response and oxidative stress in the condition of elevated glucose levels and hypoxia. This study observed that high glucose and hypoxia upregulated HIF-1α expression, while downregulated HIF-1α decreased the level of inflammation (Zhao et al., 2021). Astrocytes have been shown to participate in both innate and subsequent adaptive immune responses (Han et al., 2021). HIF-1α was identified as a mediator in the transcriptional regulation of chemokines, specifically monocyte chemoattractant proteins 1 (MCP-1/CCL2) and 5 (Ccl12), in hypoxic astrocytes (Mojsilovic-Petrovic et al., 2007). Interleukin-1 (IL-1β) beta is a mediator that triggers inflammation. Another comparative study shows that HIF-1α mediates transcriptional activation of IL-1β in astrocyte cultures which also demonstrates the association between HIF-1α and inflammation in astrocyte (Zhang et al., 2006). On the other hand, HIF-1α also plays an important role in the pathology of neuroinflammation in microglia. A study shows Aβ exposure initiates immediate microglial inflammation and this process is proved to rely on the mTOR-HIF-1α pathway (Baik et al., 2019). These pieces of evidence suggest that HIF-1α is related to inflammation. Interestingly, the interaction between HIF-1α and inflammation seems influenced by the degree of inflammation. A study by de Lemos et al. (2013) revealed that HIF-1α assumes a significant role in an acute inflammation model induced by pro-inflammatory TNF-α, IL-1β, and IFN-γ while in a chronic model of inflammation using an APP/PS1 transgenic mouse model of AD HIF-1α seems to have no effect. This phenomenon could possibly be explained by the deactivation of relevant cells following acute inflammatory responses. In fact, studies indicate that once activated by acute inflammation, microglial cells enter a state of chronic tolerance due to extensive defects in energy metabolism and subsequent attenuation of immune responses, including cytokine secretion and phagocytosis (Baik et al., 2019).

Despite numerous evidence confirming HIF-1α as a risk factor in AD, intricately linked to key mechanisms such as Aβ aggregation, tau phosphorylation, neuroinflammation, some studies suggest that HIF-1α may exhibit a protective role in the onset of AD. Desferoxamine (DFO) is a medication used to treat iron overload in conditions such as thalassemia and hemochromatosis. Research indicates that DFO has a protective effect against neurological damage caused by ischemia and hypoxia (Li et al., 2008). A comparative study suggests that inhibiting HIF-1α diminishes the protective effect of DFO, indicating that DFO’s neuroprotection involves the induction of HIF-1α (Hamrick et al., 2005). Methylene blue (MB) is another drug with neuroprotective effects. Investigations have shown that, the nuclear translocation of HIF-1α increased nearly threefold after MB treatment compared to the control group. This suggests that its protective effect may be linked to HIF-1α (Ryou et al., 2015). Due to hypoxia and ischemia being significant risk factors for various neurological diseases, the protective role of HIF-1α in hypoxia and ischemia indirectly suggests its protective effects in AD and other neurodegenerative disorders. Other studies also indicate that HIF-1α is a crucial component in several other neuroprotective pathways. Cardamonin is a chalcone with neuroprotective activity. Compelling evidence showed that in middle cerebral artery occlusion-treated mice, cardamonin reduced brain injury and stimulated the activation of the HIF-1α/VEGFA signaling and blocking the HIF-1α/VEGFA signaling with an inhibitor can reverse such protective effects. This indicates that HIF-1α is involved in neuroprotective activity (Ni et al., 2022). Due to the fact that ischemia-reperfusion injury concurrently triggers neuropathology and gene expression associated with AD, including the development of amyloid plaques, neurofibrillary tangles, and hippocampal atrophy, which are crucial for the progression of AD (Pluta and Ulamek-Koziol, 2021), the protective role of HIF-1α against ischemia-reperfusion injury indirectly suggests its protective effects against AD.

## 3 Oxidative stress and AD

Oxidative stress refers to an imbalance between the production of ROS and the body’s ability to detoxify them or repair the resulting damage. ROS are highly reactive molecules containing oxygen that can damage cells and tissues in the body. Oxidative stress can result from various factors such as environmental pollutants, toxins, poor diet, radiation, smoking, and even normal metabolic processes in the body (Lim and Thurston, 2019). Hypoxia can also lead to different pathological processes that can eventually develop into oxidative stress. Hypoxia undoubtedly has a close relationship with mitochondrial dysfunction as hypoxic condition can promote the production of ROS, cause damage to mitochondrial membrane potential (MMP) and mitochondrial DNA (mtDNA) and further lead to the insufficient energy production. Recent studies on cold inducible RNA binding protein (Cirbp) suggested that it can rescue cognitive retardation and dendritic spine injury as Cirbp can reduce the abnormal expression of PSD95, a vital synaptic scaffolding molecule, and attenuate hypoxia induced deficiency of energy and oxidative stress (Zhou et al., 2021; Liu et al., 2022). Cirbp can also control mitochondrial homeostasis and ATP biogenesis at hypoxic condition by sustaining the protein levels of respiratory chain complexes II (SDHB) and IV (MT-CO1) and directly binding 3′UTR of Atp5g3 (Liu et al., 2022). In conclusion, hypoxia is one of the most important factors leading to oxidative stress as it can induce unfavorable factors like mitochondrial dysfunction, Aβ accumulation as well as inflammation. At the same time, hypoxia can also directly lead to oxidative stress. It is reported that hypoxic conditions could upregulate HIF-1α expression and HIF-1α can regulate oxidative stress through HIF-1α/JMHD1A pathway (Zhao et al., 2021). However, there are also researches showing that HIF-1α plays a protective role against oxidative stress. Research showed the expression of mito-HIF-1α, a mitochondrial-targeted form of HIF-1α, can decrease apoptosis induced by hypoxia or H_2_O_2_ treatment (Li H. et al., 2019). Mito-HIF-1α can also reduce the production of ROS and the collapse of mitochondrial membrane potential (Li H. et al., 2019) which means it can protect mitochondria from oxidative stress and hypoxia.

When oxidative stress overwhelms the body’s antioxidant defenses, it can lead to damage to proteins, lipids, and DNA, contributing to various diseases such as cancer, neurodegenerative disorders, cardiovascular diseases, and aging. As the accurate pathogenesis of AD is still unknown, oxidative stress is supposed to play an important role in the pathogenesis of AD and is supposed to be a potential treatment target for AD. Oxidative stress is one of the major events involved in AD (Tönnies and Trushina, 2017; Dumitrescu et al., 2018; Ionescu-Tucker and Cotman, 2021). Currently, many studies on AD focus on the degeneration of neuronal cells, which can be caused by several risk factors, including oxidative stress. The damaged DNA bases, protein oxidation and lipid peroxidation products in brain after oxidative stress are sings of AD (Nunomura and Perry, 2020). Recent studies have found significant differences in 8-OHdG and 8-OHdG/2-dG between patients with mild cognitive impairment due to AD and normal elderly subjects. Both of 8-OHdG and 8-OHdG/2-dG are markers of DNA oxidative damage and can be used to assess the oxidative damage to the DNA (Peña-Bautista et al., 2019). In addition, oxidative stress also leads to the differences in protein between AD patients and normal people. Hydroxyl free radicals are known to convert phenylalanine to the non-physiological isomers of tyrosine o-tyrosine and m-tyrosine (o-Tyr and m-Tyr) (Mohás-Cseh et al., 2022). Previous research found protein oxidation (m-Tyr and o-Tyr) in AD plasma and CSF samples (Ryberg et al., 2004; Ahmed et al., 2005). Meanwhile, since 3-nitrotyrosine (3-NT) is a marker of protein oxidation, there are also studies suggesting using 3-NT as a marker for early diagnosis of AD (Ryberg et al., 2004). Herpes simplex virus type-1 (HSV-1), a DNA neurotropic virus, has been considered a potential etiological agent of AD through inducing incomplete autophagic response according to previous research (Santana et al., 2012; De Chiara et al., 2019). Oxidative stress induced by HSV-1 infection may promote the development of AD. Oxidative stress could significantly enhance HSV-1 infection-mediated intracellular Aβ accumulation and further inhibit its secretion into extracellular media (Santana et al., 2013). Additionally, studies found that at the prodromal stage of AD, there are oxidized RNAs including mRNA, rRNA, and tRNA have been identified in patients’ brains. Oxidative stress interferes with both translational machineries and regulatory mechanisms of noncoding RNAs, especially microRNAs and leads to retarded or aberrant protein synthesis (Nunomura and Perry, 2020). As one of the AD central pathological lesions in brain, NFTs are composed mainly of hyperphosphorylated tau (Naseri et al., 2019) and oxidative stress has a close relationship with tau pathology. Early studies found that specific fatty acid oxidative products could provide a direct link between oxidative stress mechanisms and the formation of NFTs in AD (Gamblin et al., 2000). All these evidence show that oxidative stress indeed takes part in the pathology of AD and oxidative damage of DNA can be an early marker of AD. Besides the direct connections, oxidative stress is also linked with AD due to some key events like Aβ accumulation, inflammation, mitochondrial dysfunction, metal dysregulation, and protein misfolding. In the next section, we will explore how the oxidative stress due to factors like mitochondrial dysfunction, DNA damage, Aβ pathology, and inflammation can be prodromal to AD.

### 3.1 Mitochondrial dysfunction and oxidative stress

Mitochondria play an important role in cells, including ATP production, intracellular Ca^2+^ regulation, ROS production, and cell damage and death (Bhatti et al., 2017; Marín et al., 2020). Research has shown that mitochondrial dysfunction is associated with AD. For example, due to the special open circular structure of mtDNA, it is highly vulnerable to oxidative damage. ROS leads to the oxidation of guanosine to form 8-OHdG and results in mtDNA mutations including base mispairing, random point mutation as well as deletions (Soltys et al., 2019; Antonyová et al., 2020) and the mutations in mtDNA are linked with misfolding and aggregation of Aβ, α-syn and tau, and neuronal apoptosis (Sengupta et al., 2015; Antonyová et al., 2020). Moreover, the connection between the impact of mtDNA haplogroups B5a on the onset of AD and mitochondrial abnormalities under oxidative stress has also been demonstrated (Bi et al., 2015). Mitochondrial dysfunction has connection with ROS accumulation. Early-onset Alzheimer’s disease (EOAD) refers to AD that develops in individuals under the age of 65. Clinical studies have implied that changes in mtDNA methylation and transitions (position 5633: T → C; position 7476T: C → T; and position 15812A: G → A) can play a significant role in the development of EOAD (Chagnon et al., 1999). Thus, considering the relationship between AD and mitochondrial dysfucntion, if there is evidence showing that mitochondrial dysfunction is related to oxidative stress, we can infer that AD is also related to oxidative stress.

**FIGURE 3:**
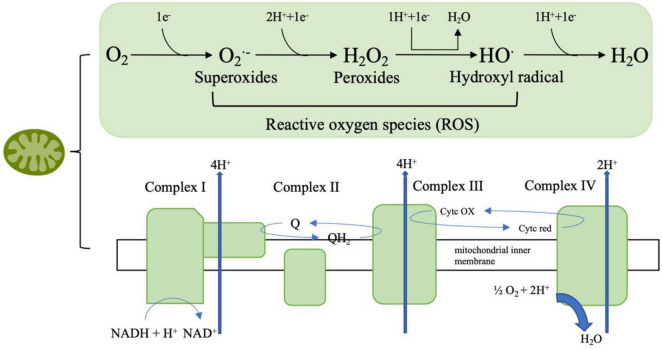
The mitochondrial respiratory chain involves a series of protein complexes (I–IV) that transfer electrons from NADH and FADH_2_ to oxygen, thus creating an electrochemical gradient that pumps protons across the inner mitochondrial membrane. This gradient drives ATP synthesis through ATP synthase. During this process, molecular oxygen can be partially reduced to form reactive oxygen species (ROS) such as superoxide (O_2_⋅^–^), hydrogen peroxide (H_2_O_2_), and hydroxyl radicals (HO⋅), which are eventually converted to water (H_2_O).

Mitochondria are the primary site of ROS which are closely associated with oxidative stress. Therefore, it can be inferred that mitochondrial dysfunction is related to oxidative stress. ROS species are the product of one-electron reduction of oxygen and include singlet oxygen (1O_2_), superoxides (O_2_⋅^–^), peroxides (H_2_O_2_), hydroxyl radical (⋅OH), and hypochlorous acid (HClO) (Taysi et al., 2019; Sahoo et al., 2022; Figure 3). Complexes I and III of the mitochondrial respiratory chain are the major sited of superoxide production (Mailloux and Harper, 2011). As a by-product of cellular metabolism, ROS has both beneficial and deleterious effects on our health. On one hand ROS contribute to the healthy cell function as they play an essential role in the regulation of growth, apoptosis, autophagy, memory, blood pressure, cognitive function as well as immune function (Scherz-Shouval et al., 2007; Oswald et al., 2018; Luo et al., 2019; Lloberas et al., 2020). Also, recent research showed that ROS has antimicrobial activity against Gram-positive and Gram-negative viruses as well as fungi (Dryden, 2018) and play a role in modulating endoplasmic reticulum and Golgi homeostasis (Mennerich et al., 2019). However, at high concentrations, ROS are harmful for living organisms as ROS are included in processes of many diseases like metabolic disorders, genetic diseases, diabetes, cancer as well as neurodegenerative diseases. The accumulation of ROS is related to lipid peroxidation, protein oxidation and DNA damage which are all features of oxidative stress (Su et al., 2019; Juan et al., 2021).

Furthermore, oxidative stress can induce damage to mitochondria through ROS which is characterized by the cytochrome c (cyt C) release and the increase in mtDNA fragmentation (Rizwan et al., 2020).

Cytochrome c is a small heme protein associated with the inner membrane of the mitochondria. It plays a crucial role in the electron transport chain, transferring electrons between Complex III and Complex IV. cyt C is closely linked to the production of ROS in the mitochondria. During the electron transport chain, electrons are transferred through various complexes, including cyt C. If the electron transfer process is inefficient, electrons can prematurely reduce oxygen, leading to the formation of O_2_⋅^–^, a type of ROS. Additionally, when cyt C is released into the cytosol during apoptosis, it can enhance ROS production, which further contributes to cellular damage and the progression of cell death (Radi et al., 2014; Cadenas, 2018). Thus, considering cyt C is one of the important part of mitochondria, we can infer that mitochondria dysfunction has a connection with oxidative stress. On the other hand, mtDNA is highly susceptible to ROS influence due to its attachment with inner membrane and the lack of protective histones or nonhistone proteins. Evidence shows that when exposed directly to ROS, the levels of oxidative mtDNA damage are higher and more extensive compared to nuclear DNA (Richter et al., 1988). There is also a vicious cycle theory of mitochondrial ROS production which proposes a self-perpetuating cycle where mitochondrial dysfunction leads to increased ROS production. This elevated ROS generation, in turn, causes further damage to mitochondrial components, exacerbating dysfunction and ROS production (Bandy and Davison, 1990).

### 3.2 Aβ and oxidative stress

Alzheimer’s disease is a progressive neurodegenerative condition characterized by the accumulation of extracellular Aβ plaques and intracellular neurofibrillary tangles composed of hyperphosphorylated tau-protein in specific regions of the human brain, notably cortical and limbic areas. Clinical signs include memory impairment and deteriorating neurocognitive function. Dysregulated processing of APP by β-secretases and γ-secretases results in the generation of Aβ40 and Aβ_42_ monomers, which subsequently aggregate to form senile plaques (Soria Lopez et al., 2019; Tiwari et al., 2019). Given the relationship between Aβ and AD, and considering the compelling evidence indicating the involvement of Aβ in oxidative stress, it can be inferred that oxidative stress is associated with AD.

Amyloid-beta has indeed been proven to be associated with oxidative stress in AD pathogenesis and progression (Tamagno et al., 2012; Hilt et al., 2018; Mcdonald et al., 2021), both *in vivo* and *in vitro* investigation.

Electron paramagnetic resonance (EPR), also known as electron spin resonance (ESR), is a technique which is particularly useful in studying paramagnetic species, like free radicals (Dikalov et al., 2018; Sahu and Lorigan, 2020). Aβ_1–40_ and Aβ_25–35_ fragments refer to specific shorter protein of the full Aβ peptide sequence. Studies have found that they participated in the pathology of AD and related with other neurodegenerative disorders (Kaminsky et al., 2010; Yang et al., 2020). Spin trapping experiments conducted by Butterfield et al. (1994) employing highly purified N-tert-butyl-phenylnitrone (PBN) revealed that both Aβ_1–40_ and Aβ_25–35_ autonomously prompted the transformation of PBN from its non-paramagnetic nitrone form to the stable, paramagnetic nitroxide form in phosphate buffered saline (PBS), and the reaction only achievable through interaction with a free radical (Butterfield et al., 1994; Hensley et al., 1995). Comparable to Aβ_1–40_, Aβ_1–42_ was likewise demonstrated to produce an EPR signal within this framework. Protein carbonyl and 3-NT are indicators of protein oxidation (Campolo et al., 2020; Akagawa, 2021). Studies *in vitro* also find Aβ_25–35_, Aβ_1–40_, and Aβ_1–42_ were demonstrated to cause notable rises in protein carbonyls as well as 3-NT in cortical synaptosomes, cultured hippocampal neurons, primary neuronal cultures, and cultured astrocytes (Harris et al., 1995a,b; Yatin et al., 1998). When it comes to *in vivo* researches, studies suggest that Aβ increases the generation of ROS in neurons through activation of the NADPH oxidase. Moreover, Aβ induces mitochondrial depolarization through calcium overload and free radical production, which can ultimately lead to oxidative damage and trigger cell death via the opening of the mitochondrial permeability (Angelova and Abramov, 2017; Chauhan and Chauhan, 2020). AD patients show an abnormal level of brain metals including copper, zinc, iron, calcium, and aluminum (Tong et al., 2018; Huat et al., 2019; Mcdonald et al., 2021; Peng et al., 2021; Plascencia-Villa and Perry, 2021), the incorrect accumulation of the metal in different brain regions induces oxidative stress (Wang et al., 2020). Evidence indicate that Aβ plays a significant role in metal-induced oxidative stress in AD patients. The binding of iron to Aβ can induce misfolded Aβ aggregating, leading to the formation of neurotic plaques and can also increase neurotoxicity of Aβ (Rogers et al., 2019). At the same time Aβ can be a pro-oxidant, and when it is complexed with copper or iron, it can produce ROS (H_2_O_2)_ by redox activity and causes oxidative damage to protein and lipid (Greenough et al., 2013; Wang et al., 2020). Different mentals may have distinct roles in AD pathology. The role of copper as a risk factor for AD has been confirmed to promote the aggregation of Aβ *in vitro*. Additionally, copper enhances APP translation via the 5′ untranslated region (5′ UTR) of mRNA in SH-SY5Y cells, and increases amyloidogenic processing and the expression of related pro-inflammatory cytokines (such as MCP-5) in Alzheimer’s APP/PS1 double transgenic mice (Yang et al., 2019). Zinc, on the other hand, performs a protective effect as zinc can exchange with copper in the Aβ-copper complex and protect cells from oxidation (Vašák and Meloni, 2017). Studies have found that antioxidants can improve AD symptoms by alleviating oxidative stress, and Aβ is involved in this process. Nobiletin is a bioflavonoid isolated from citrus fruits peels with anti-oxidative function. In a study, rats were treated with nobiletin after bilateral intrahippocampal (CA1 subfield) injection of Aβ_1–40_. Research found that mice treated with nobiletin performed better in behavioral observations and memory performance compared to the control group. Nobiletin treatment was also associated with lower hippocampal levels of ROS and partial reversal of superoxide dismutase (SOD) activity (Ghasemi-Tarie et al., 2022). These findings indicated that nobiletin prevents Aβ_1–40_-induced AD via the inhibition of oxidative stress. N-adamantyl-4-methylthiazol-2-amine (KHG26693) is a new thiazole derivative and was reported that it effectively inhibits Aβ-induced oxidative damage in primary cortical neuron cultures (Cho et al., 2016). In a recent study, malondialdehyde (sign of lipid oxidation) and protein carbonyl (sign of protein oxidation) levels increased in the Aβ-treated group and were significantly downregulated by KHG26693 treatment. Meanwhile KHG26693 significantly decreased Aβ-induced ROS generation by 47% compared to the control group (Kim et al., 2017). These results illustrate that KHG26693’s protection against Aβ-induced oxidative stress stems from its ability to restrain the excessive generation of ROS triggered by Aβ. Other antioxidants like benzothiazole, azelnidipine, engeletin, adenosine, and epigallocatechin gallate have the similar effect of ameliorating Aβ-related oxidative stress by different pathways including Keap1/Nrf2 pathway, NFκB pathway, and ERα pathway, etc and have a potential therapeutic efficacy in AD (Cifelli et al., 2016; Zhang et al., 2017; Teng et al., 2019; Huang et al., 2020; Zeng et al., 2022).

The studies here discussed demonstrated that Aβ is associated with oxidative stress. Therefore, we can infer that oxidative stress is related to AD due to its role on Aβ production and deposition.

### 3.3 Inflammation and oxidative stress

**FIGURE 4:**
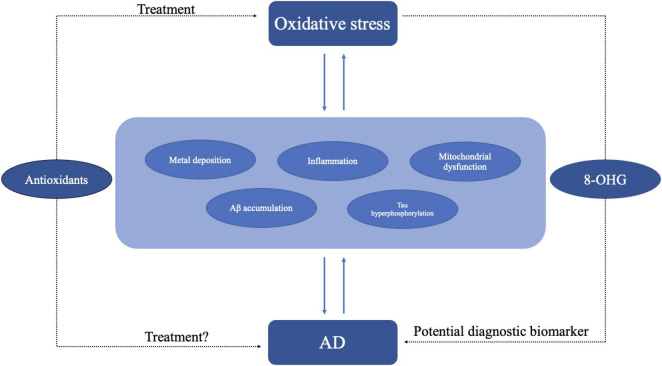
Oxidative stress may lead to AD through different pathogenesis including inflammation, metal deposition, mitochondrial dysfunction, and Aβ accumulation. In turn AD itself may exacerbate oxidative stress. Antioxidant might be an effective treatment for oxidative stress. As oxidative stress plays an important role in AD, the use of antioxidant molecules is also a potential treatment for AD. Oxidative stress products like 8-OHG could be diagnostic biomarkers of AD.

Inflammation is a physiological response to harmful stimuli, such as pathogens, damaged cells, or irritants. It is a protective mechanism that aims to remove the harmful stimuli and initiate the healing process. There are two stages of inflammation, acute and chronic inflammation. While acute inflammation is typically a short-term and beneficial response, chronic inflammation can contribute to various diseases, including arthritis, cardiovascular diseases, and cancer (Hussain and Harris, 2007; Lin and Karin, 2007). Numerous research and studies highlight that both systemic inflammation and neuroinflammation are tightly connect with the pathology of AD (Doifode et al., 2021; Twarowski and Herbet, 2023). Specifically, neuroinflammation denotes an inflammatory reaction occurring within the central nervous system (CNS), triggered by diverse pathological insults such as infection, trauma, ischemia, and toxins. This cascade entails the release of pro-inflammatory cytokines like IL-1β, IL-6, IL-18, and tumor necrosis factor (TNF), chemokines including C-C motif chemokine ligand 1 (CCL1), CCL5, and C-X-C motif chemokine ligand 1 (CXCL1), as well as small-molecule mediators like prostaglandins and nitric oxide (NO) by innate immune cells in the CNS. Microglia and astrocytes orchestrate this response principally (DiSabato et al., 2016; Leng and Edison, 2021). Research on neuroinflammation suggests that microglia, astrocytes, and neurons collaborate to drive neurodegeneration in a coordinated manner. Studies have demonstrated that Aβ activates the NF-κB pathway in astrocytes, leading to heightened complement C3 release. Subsequently, C3 acts on C3a receptors present on neurons and microglia, culminating in neuronal dysfunction and microglial activation (Lian et al., 2015). Conversely, activated microglia have been found to induce neurotoxic astrocytes by secreting IL-1α, C1q, and TNF. This interplay between microglia and astrocytes may establish a positive feedback loop in AD, perpetuating an uncontrolled and self-amplifying inflammatory response (Leng and Edison, 2021). Additionally, aberrant neuronal-glial communication has been observed in AD. Under normal circumstances, neuronal-microglial communication via CD200-CD200R and CX3CL1-CX3CR1 (microglial receptor) signaling pathways help maintaining microglial homeostasis. However, reduced expression of CD200, CD200R, and CX3CR1 in the brains of individuals with AD suggests a loss of regulatory control over microglial behavior (Bolós et al., 2017). When it comes to systemic inflammation, cross-sectional investigations reveal that individuals with cognitive impairment showed heightened systemic inflammation and elevated microglial activation compered to cognitively healthy subjects (Surendranathan et al., 2018). There are also evidences showing that gut inflammation can affect AD pathology through gut-brain axis (Abdel-Haq et al., 2019; Doifode et al., 2021). As discussed earlier, both neuroinflammation and systemic inflammation are linked to AD. Therefore, demonstrating the association between inflammation and oxidative stress indirectly implies a connection between oxidative stress and the occurrence of AD. Inflammation and oxidative stress seem like two indivisible parts in a large number of diseases including cardiovascular diseases, cancer, and AD (Chamorro et al., 2016; Marchev et al., 2017; Papaconstantinou, 2019; Li et al., 2020; Figure 4). Hypoxia is a common feature in inflammation and can lead to chronic inflammation through the activation of NF-κB and multiple isoforms of HIFs and PHDs (Watts and Walmsley, 2019; Korbecki et al., 2021). A treatment that may regulate both inflammation and oxidative stress at the same time is the inhibition of TREM1 (Li Z. et al., 2019). Moreover, mounting evidence suggest that continued oxidative stress can lead to chronic inflammation by activating a variety of transcription factors like NF-κB, AP-1, p53, HIF-1α, PPAR-γ, β-catenin/Wnt, and Nrf2 (Reuter et al., 2010; Fan et al., 2013; Korbecki et al., 2021). These transcription factors link inflammation to diseases through different signaling pathways. For example, NF-κB and STAT3 are rapidly activated in response to diverse stimuli, including oxidative stress. Upon activation, they govern the expression of genes involved in anti-apoptotic functions, proliferation, and immune responses. Some of these genes overlap, requiring transcriptional cooperation between the two factors. The activation and interplay of STAT3 and NF-κB are pivotal in regulating the interaction between malignant cells and their microenvironment, particularly with inflammatory and immune cells infiltrating tumors (Grivennikov and Karin, 2010; Fan et al., 2013). Metals also play an important role in inflammation. Zinc, as an essential micronutrient, is involved in both inflammation and oxidative stress. The lack of Zinc can elevate inflammatory response as it is involved in the NF-κB pathway (Gammoh and Rink, 2017). Chronic inflammation manifests through heightened production of inflammatory cytokines. In Certain conditions like obesity which correlates with chronic inflammation, individuals with inadequate zinc intake exhibit reduced plasma and intracellular zinc levels, coupled with elevated gene expression of IL-1α, IL-1β, and IL-6, in contrast to those with sufficient zinc intake (Gammoh and Rink, 2017). As critical participants in inflammation, microglia and astrocytes can be regulated by ROS as well as by pro-inflammatory molecules such as MAPK and NF-κB pathway and other pathways (Park et al., 2015). On the other hand, microglia and astrocytes can release pro-inflammatory molecules like cytokines and ROS (Chen and Zhong, 2014). In addition, microglia and astrocytes interact with Aβ. The accumulation of neurotoxic Aβ itself can be regulated by microglia and their receptors as microglia can phagocyte Aβ (Ennerfelt et al., 2022), while Aβ can activate both microglia and astrocytes and be deposited in brain thus leading to ROS production: this in turn may result in oxidative stress and potentially lead to AD and other neurodegenerative disease (Chen and Zhong, 2014). A recent study revealed a critical function of SYK signaling in microglia as it can impede the development of disease-associated microglia, alter AKT/GSK3β-signaling and restrict Aβ phagocytosis by microglia (Ennerfelt et al., 2022). The strong correlation between inflammation and oxidative stress is further evidenced by studies demonstrating aggravated inflammatory phenotype in the absence of antioxidant defense proteins, such as superoxide dismutases, heme oxygenase-1, and glutathione peroxidases or overexpression of ROS producing enzymes, for example, NADPH oxidases (Steven et al., 2019).

## 4 Antioxidants are potential treatments for AD

As AD has caused great social burden, there is an urgent need for new therapeutic targets and approaches. Efforts concentrating on lowering amyloid beta or hyperphosphorylated Tau protein have mostly proven unsuccessful in clinical trials. As emerging evidence shows oxidative stress may be one of the key mechanisms of AD, the antioxidants have become a potential treatment for AD (Ionescu-Tucker and Cotman, 2021; Figure 4). Walnuts, one of the most common antioxidative foods in our daily life, can reduce oxidative stress by decreasing the generation of free radicals and has a beneficial effect on memory, learning, anxiety (Chauhan and Chauhan, 2020). Researches on the APP-transgenic AD mouse model have shown that mice with a walnut-based diet had an improvement in antioxidant defense and significant reductions in free radicals’ levels, lipid peroxidation and protein oxidation compared with a control diet (Chauhan and Chauhan, 2020). Recent studies on PTEN-induced putative kinase 1 (PINK1) show that PINK1 overexpression can reverse the abnormal changes in mitochondria dynamics, defective mitophagy and decreased ATP levels in the hippocampus (Du et al., 2017). PINK1 overexpression activates Nrf2 signaling increases the expression of antioxidant proteins and reduces oxidative damage. PINK1 can also alleviate tau hyperphosphorylation through PI3K/Akt/GSK3β signaling (Wang et al., 2022). As an electron and proton carrier, Coenzyme Q10 (CoQ10) or ubiquinone plays an important part in mitochondrial bioenergetics and has long been used as antioxidant and mitochondrial energizer (Garrido-Maraver et al., 2014; Li X. et al., 2019; Alimohammadi et al., 2021). Recent studies showed CoQ10 can protect cells from lipid peroxidation-mediated cell death thus potentially reducing oxidative stress and conferring neuroprotective properties (Arslanbaeva et al., 2022). UBIA prenyltransferase domain-containing protein 1 (UBIAD1) which is responsible for the biosynthesis of non-mitochondrial CoQ10 has a similar effect like CoQ10 (Arslanbaeva et al., 2022).

## 5 Conclusion

This review primarily summarizes the impact of hypoxia on AD focusing on two aspects: HIF-1α and oxidative stress. Hypoxia is the direct and the most important consequence of both HIF-1α release and oxidative stress. These two phenomena are linked to pathological hallmarks of AD like the aggregation of Aβ, mitochondrial dysfunction and tau accumulation, thus suggesting that hypoxia is linked to AD. Current research indicates that HIF-1α has a relationship with Aβ pathology of AD. HIF-1α can increase Aβ production by regulating the expression of BACE-1. Moreover, recent studies have found an interaction between HIF-1α and γ-secretase. HIF-1α not only binds to γ-secretase but also activates it, promoting the production of Aβ under hypoxic conditions and reducing cerebral blood flow. Additionally, HIF-1α plays a crucial role as a key regulator of Aβ generation under the influence of high glucose and other environmental factors. Specifically, under high glucose conditions, increased ROS levels trigger the activation of HIF-1α and LXRα/ABCA1. This stimulation leads to lipid raft rearrangement, enhancing the production of Aβ mediated by BACE1. HIF-1α is also associated with tau phosphorylation and neuroinflammation as it can lead to a deficiency in LCMT1 as well as PP2A. Also, HIF-1α mediates neuroinflammatory responses in microglial cells through mTOR-HIF-1α pathways also involving Aβ. Moreover, compelling studies have provided evidence of a protective role for HIF-1α in neurodegenerative diseases like AD. For example, inhibiting HIF-1α diminishes the protective effect of DFO, which plays a crucial role in managing iron metabolism disorders and has a protective effect against neurological damage caused by ischemia and hypoxia (Li et al., 2008). This indicates that HIF-1α might not only serve as a potential target for AD drugs but also potentially plays a direct protective role to AD and other neurodegenerative disease. However, further investigations are needed to confirm our hypothesis. Oxidative stress interacts with various AD risk factors, including mitochondrial dysfunction, Aβ aggregation, and neuroinflammation. Studies found that protein oxidation (m-Tyr/Phe and o-Tyr/Phe) in AD plasma and CSD samples which confirms oxidative stress is associated with the development of AD. Also, we explored the possibility of using antioxidants as potential therapeutic agents for AD. Efforts aimed at lowering amyloid beta or hyperphosphorylated Tau protein have mostly proven unsuccessful in clinical trials. Emerging evidence suggests that oxidative stress may be one of the key mechanisms of AD, therefore, the antioxidants emerge as potential treatments for AD, thus prompting further investigations.

## Author contributions

MC: Writing – review & editing. BT: Writing – original draft. WG: Writing – original draft. CX: Writing – original draft. ZM: Writing – original draft. JM: Writing – review & editing.
